# The Matricellular Receptor LRP1 Forms an Interface for Signaling and Endocytosis in Modulation of the Extracellular Tumor Environment

**DOI:** 10.3389/fphar.2015.00271

**Published:** 2015-11-10

**Authors:** Bart Van Gool, Stéphane Dedieu, Hervé Emonard, Anton J. M. Roebroek

**Affiliations:** ^1^Laboratory for Experimental Mouse Genetics, Department of Human Genetics, KU Leuven, Leuven, Belgium; ^2^Centre National de la Recherche Scientifique, Unité Mixte de Recherche 7369 Matrice Extracellulaire et Dynamique Cellulaire, Université de Reims Champagne-Ardenne, Unité de Formation et de Recherche Sciences Exactes et Naturelles, Reims, France

**Keywords:** LRP1, cancer, extracellular matrix, metastasis, signaling, endocytosis

## Abstract

The membrane protein low-density lipoprotein receptor related-protein 1 (LRP1) has been attributed a role in cancer. However, its presumably often indirect involvement is far from understood. LRP1 has both endocytic and signaling activities. As a matricellular receptor it is involved in regulation, mostly by clearing, of various extracellular matrix degrading enzymes including matrix metalloproteinases, serine proteases, protease inhibitor complexes, and the endoglycosidase heparanase. Furthermore, by binding extracellular ligands including growth factors and subsequent intracellular interaction with scaffolding and adaptor proteins it is involved in regulation of various signaling cascades. LRP1 expression levels are often downregulated in cancer and some studies consider low LRP1 levels a poor prognostic factor. On the contrary, upregulation in brain cancers has been noted and clinical trials explore the use of LRP1 as cargo receptor to deliver cytotoxic agents. This mini-review focuses on LRP1’s role in tumor growth and metastasis especially by modulation of the extracellular tumor environment. In relation to this role its diagnostic, prognostic and therapeutic potential will be discussed.

## Introduction

The matricellular receptor low-density lipoprotein (LDL) receptor-related protein 1 (LRP1) is a multifunctional receptor implicated in both endocytosis and signaling pathways (Lillis et al., 2008). Numerous ligands, both structurally and functionally diverse, bind to LRP1 and the endocytosis of many of these ligands is coupled to activation of signal pathways. Together with its broad expression pattern, the multifunctionality of this receptor accounts for its involvement in various physiological and pathological processes including extracellular matrix modulation, transport across the blood–brain barrier (BBB), coagulation, inflammation, Alzheimer’s disease, atherosclerosis, etc. The role of LRP1 in many of these processes is discussed in detail in recent reviews (Kanekiyo and Bu, 2014; Strickland et al., 2014). Following upon a short general description of the structure and the function of LRP1, the present mini-review, however, focuses on the often indirect role of LRP1 in tumor growth and metastasis by modulation of the extracellular tumor environment.

## General Role of LRP1 in Endocytosis and Cell Signaling

Lipoprotein receptor related-protein 1, a type I transmembrane receptor, is a member of the LDL-receptor gene family (Lillis et al., 2008). The LRP1 precursor is cleaved by furin in the *trans*-Golgi to generate a 515 kDa N-terminal α-subunit and an 85 kDa C-terminal β-subunit. In the mature two-chain structure, the entirely extracellular α-subunit, containing the ligand binding domains, is non-covalently linked to the transmembrane-containing β-subunit. After maturation, arrival at the cell surface and ligand binding it undergoes highly efficient constitutive endocytosis via clathrin-coated pits and recycling. The dominant signals for endocytosis are YxxL and dileucine motifs in the cytoplasmic or intracellular domain of the β-subunit (Li et al., 2000), whereas two NPxY motifs, of which the latter overlaps with the YxxL motif, are secondary endocytosis signals and binding sites for adaptor proteins involved in signaling (Trommsdorff et al., 1998; Li et al., 2000; Loukinova et al., 2002). Analyses of knock-in mice and derived MEFs carrying inactivating mutations of the proximal NPxY and the distal NPxYxxL motifs revealed that, besides for endocytosis and signaling, these motifs are also relevant for slow recycling of LRP1 from the perinuclear compartment to the plasma membrane and even for early steps in LRP1 biosynthesis, preventing premature proteasomal degradation of precursor LRP1 (Roebroek et al., 2006; Gordts et al., 2009, 2012; Reekmans et al., 2010).

**FIGURE 1:**
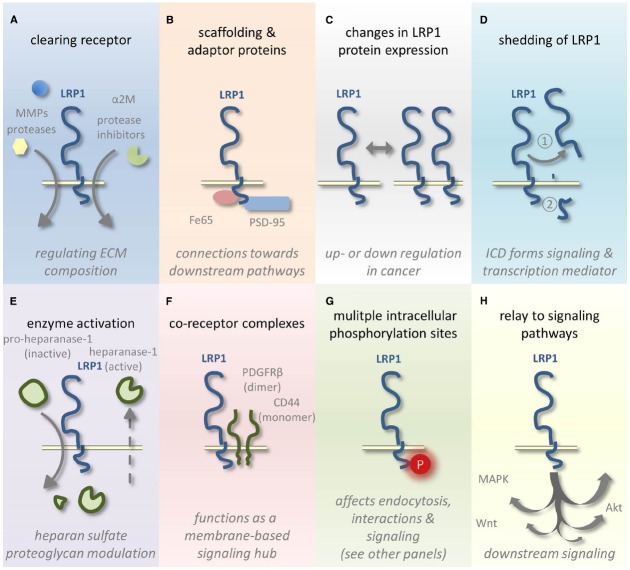
**Schematic representation of LRP1-mediated tumor growth and metastasis fine tuning. (A)** LRP1 clears various cancer-related ligands from the ECM by endocytosis. **(B)** The LRP1 ICD also interacts with several adaptor and scaffolding proteins. **(C)** LRP1 expression levels vary among different tumor types and tumor stages and **(D)** the receptor can undergo shedding and subsequent release of the ICD. **(E)** Heparanase-1 activation is affected by LRP1-mediated uptake of its inactive precursor. **(F)** The formation of co-receptor complexes with LRP1 influences signaling and **(G)** also the phosphorylation of the LRP1 ICD influences signaling and regulates endocytosis. **(H)** LRP1-mediated signaling affects several well-known pathways linked to cancer.

Lipoprotein receptor related-protein 1 ligands include proteases, protease inhibitor complexes, extracellular matrix proteins, growth factors, toxins, and viral proteins (Lillis et al., 2008). Via clearing of proteases, like (matrix-)metalloproteinases and other secreted proteins, like coagulation FVIII, LRP1 contributes to the homeostasis of many secreted proteins and the integrity of the extracellular matrix (Figure 1A). LRP1 regulates, however, also the abundance of many other proteins, including receptors present at the plasma membrane. For example, the urokinase-type plasminogen activator (uPA)-plasminogen activator inhibitor-1 (PAI-1) complex is a bivalent ligand, which triggers urokinase receptor (uPAR) internalization and regulates the uPAR signaling by bridging extracellularly uPAR and LRP1 (Gonias et al., 2011). Fe65 and PSD-95 are intracellular adaptor proteins (Figure 1B) that interconnect LRP1 to β-amyloid precursor protein (β-APP; Pietrzik et al., 2004) and *N*-methyl-D-aspartate (NMDA) receptor (May et al., 2004; Martin et al., 2008) respectively, stimulating APP endocytosis and amyloid (Aβ) generation (Pietrzik et al., 2004), and extracellular signal-regulated kinase 1/2 (ERK1/2) signaling (Martin et al., 2008).

## LRP1 and Cancer: A Long but Difficult Marriage

Lipoprotein receptor related-protein 1 has already been attributed a role in cancer shortly after its discovery in 1988 (Herz et al., 1988). Initially, several groups reported decreased LRP1 expression (Figure 1C) levels in various cancer cell lines and tissues, thus assigning a tumor suppressive role to this receptor (Kancha et al., 1994; de Vries et al., 1996; Gilardoni et al., 2003). These findings provided a rationale for earlier studies in which decreased binding and uptake of α_2_-macroglobulin (α_2_M), an LRP1 ligand, were observed in multiple cancer cell lines (Van Leuven et al., 1979; Saksela et al., 1981, 1984; Jensen et al., 1989). It should be noted, however, that under normoxia cell culture conditions cancer cell lines *in vitro* might show a reduction in LRP1 expression compared to hypoxic conditions (Montel et al., 2007). As in many tumors *in vivo* hypoxic conditions exist, this observed decrease in LRP1 expression should be interpreted with caution. Nonetheless, more recent work supports a reduction in LRP1 expression in cancer. Amos et al. (2007) compared LRP1 expression between low-grade astrocytoma and high-grade astrocytoma (glioblastoma). They correlated a decrease in LRP1 expression with more advanced tumor grade and enhanced uPA-dependent cell invasion. Previously however, Yamamoto et al. (1997) and Baum et al. (1998) have described opposite results: LRP1 expression was predominantly detected in glioblastoma and to a lesser extent in lower grade astrocytomas. *In vitro*, LRP1 expression appears to vary substantially among different glioblastoma cell lines (Maletinska et al., 2000). In hepatocellular carcinoma, colorectal carcinoma and lung adenocarcinoma, reduced LRP1 expression levels were linked to a poor prognosis and more advanced tumor stages (Obermeyer et al., 2007; Meng et al., 2011; Huang et al., 2012). Recently, it was shown that LRP1 acts in response to ApoE as an endogenous suppressor of the metastatic phenotype in melanoma (Pencheva et al., 2012). However, contrasting evidence exists suggesting a role for LRP1 in supporting thyroid and breast cancer cell invasion and metastasis (Chazaud et al., 2002; Montel et al., 2007; Dedieu et al., 2008; Fayard et al., 2009). Moreover, increased LRP1 expression was found to be predictive of more aggressive tumor behavior and associated with higher histological grade in endometrial carcinomas (Catasus et al., 2011).

Post-translational regulation of LRP1 by proteolytic cleavage (also named shedding) is a critical mechanism in regulating cell-surface LRP1 expression, especially in tumor context (Figure 1D). Since the first identification of the extracellular part of LRP1 (LRP1-ECD) solubilized in human plasma (Quinn et al., 1997), proteolytic enzymes from different classes have been identified as LRP1 sheddases (Etique et al., 2013). These include metalloproteinases such as MT1-MMP and ADAM-10 and -12, the serine proteinase tPA and BACE-1. Shedding of LRP1-ECD allows the release from the plasma membrane by γ-secretase of the intra-cytoplasmic domain of LRP1 (LRP1-ICD), which could act as signaling mediator (May et al., 2002). Accumulation of extracellular proteolytic activities associated to the tumor microenvironment could explain at least in part why cell-surface LRP1 is generally found decreased in advanced tumors. However, the significance of LRP1 shedding is not really understood in the field of malignant diseases.

Only a few *LRP1* polymorphisms or mutations were identified in cancer specimens. Benes et al. (2003) associated the C766T polymorphism with an increased risk to develop breast cancer in Caucasian women. Although this change into a thymine nucleotide does not result in an amino acid substitution, this silent mutation has previously also been linked to Alzheimer’s (Kolsch et al., 2003) and coronary artery disease (Pocathikorn et al., 2003) but also conflicting data were published (Benes et al., 2001; Pritchard et al., 2005). Recently, a LRP1-SNRNP25 fusion gene was identified in two osteosarcomas (Yang et al., 2014). Only the first eight exons including the promoter region of LRP1 are implicated in the fusion gene. Although the relevance of LRP1 expression to osteosarcoma is currently unknown, *in vitro*, however, LRP1-SNRNP25 promotes invasion and migration. LRP1-SNRNP25 expression was increased in both tumors via the LRP1 promoter activity of the fusion gene compared to the wild-type SNRNP25 expression in other osteosarcomas specimen.

## A Multitude of Cancer-Modifying Pathways

Remodeling of the ECM is essential for both tumor growth and metastasis. As a matricellular receptor, LRP1 is involved in the regulation of several ECM modifying pathways.

(Matrix)-metalloproteinases (MMPs) are key enzymes in physiological but also in cancer-related modulation of ECM and basement membrane components. Their proteolytic function mostly results in inactivation or degradation of many of their different substrates. MMPs are, however, also found involved in signaling functions in a non-proteolytic manner (Kessenbrock et al., 2010, 2015; Yamamoto et al., 2015). LRP1 mediates endocytosis of MMP-2, -9, -13, ADAMTS-4 and ADAMTS-5 and clears these proteases from the ECM (Emonard et al., 2005; Yamamoto et al., 2014, 2015). Endocytosis by LRP1 can depend on complex formation: (pro)MMP-2:TSP-2 (thrombospondin-2), proMMP-2:TIMP-2 (tissue inhibitor of metalloproteinases 2), and proMMP-9:TIMP-1 complexes are all ligands to LRP1 and cleared by this receptor (Emonard et al., 2005; Yamamoto et al., 2015). Furthermore, other MMPs are being regulated by LRP1, although indirectly, via the clearance of TIMP-1, -2, and -3 by LRP1 whether bound to an MMP (Emonard et al., 2005; Yamamoto et al., 2015) or alone (TIMP-1 and -3; Scilabra et al., 2013; Thevenard et al., 2014). These TIMPs also display signaling functions via the ERK and Wnt pathways (Liu et al., 2003; Egea et al., 2012). Also the broad spectrum protease inhibitor α_2_M binds to LRP1 followed by subsequent internalization (Andersen et al., 2000). Not only metalloproteinases are a target of this glycoprotein but also serine-, carboxyl-, and thiol proteinases are blocked from interacting with their respective substrates (Rehman et al., 2013). Besides its activity as a protease inhibitor, α_2_M was recently shown to stimulate angiogenesis via activation of stem cells through FGF-2 and nitric oxide via LRP1-mediated signaling (Sauer et al., 2013).

Heparanase-1 is another matrix modifying enzyme that is endocytosed by LRP1, both for its activation and clearance (Figure 1E). This enzyme cleaves heparan sulfate proteoglycans (HSPG), one of the core components of the ECM (Ilan et al., 2006). HSPGs not only play a role in the integrity of the ECM but also act as a storage depot for growth factors, chemokines, cytokines and enzymes. Heparanase-1 is synthesized as an inactive precursor. Activation requires proteolytic cleavage that is partly dependent on LRP1-mediated pro-heparanase-1 internalization (Figure 1E; Vreys et al., 2005). Also mature heparanase-1 can be endocytosed by LRP1 targeting it for degradation or recycling (Vreys and David, 2007).

uPA-uPAR signaling is another migration- and invasion-related pathway regulated by LRP1 that can promote cell invasion and migration (Webb et al., 2000; Amos et al., 2007; Gonias et al., 2011). uPA and tPA proteinase activity are implicated in the plasminogen activator system and as such mediate plasmin-dependent degradation of ECM proteins (Gonias et al., 2011). Interaction of uPA with PAI-1 on uPAR stimulates uPAR-LRP1 complex formation and subsequent endocytosis (Czekay et al., 2001). This affects uPAR presence at the plasma membrane with consequences for ECM degradation via the plasminogen activation system and uPAR-integrin interaction, both important for cell migration. Also for angiogenesis the uPA-plasmin system is highly relevant (Raghu et al., 2010). Furthermore, LRP1 was shown to promote maturation of the integrin β1 precursor thereby increasing the level of integrin β1 at the cell surface (Salicioni et al., 2004). LRP1 also binds to α_M_β_2_ thereby altering integrin function. In macrophages, LRP1 is important for α_M_β_2_ internalization thereby possibly influencing macrophage-mediated inflammation (Ranganathan et al., 2011).

Migration of malignant cells is further affected by LRP1-CD44 complexes in the cell membrane (Figure 1F). LRP1 was recently shown to control the adhesion in tumor cells via interaction with, and internalization of CD44, a transmembrane glycoprotein (Perrot et al., 2012). CD44 mediates cell adhesion to the ECM, migration and is probably involved in tumor and metastasis initiation. Like LRP1, CD44 acts as an interface for signal transduction at the cell surface as recently reviewed (Orian-Rousseau, 2015). A lowering in LRP1 expression as observed in certain cancers (see supra) could thus result in CD44 accumulation at the cell surface and enforced cancer cell attachment.

Besides this, probably far from complete, overview of LRP1-related ECM modifying processes, LRP1 also forms co-receptor complexes (Figure 1F) at the cell surface with receptors involved in cancer-related pathways. A good example is the association between LRP1 and the platelet-derived growth factor receptor-β (PDGFR-β). LRP1 not only mediates PDGF internalization and degradation, in two accompanying papers, PDGF-BB was shown to mediate the phosphorylation (Figure 1G) of the Tyr_63_ in the distal NPxY motif of LRP1 located in caveolae (Boucher et al., 2002; Loukinova et al., 2002). This process is dependent on PDGFR activation and on the kinase activity of the c-Src family of proto-oncogenic tyrosine kinases. This relationship links LRP1 to Ras, c-Myc, MAPK, and Akt/PI3K signaling, well known pathways implicated in oncogenesis (Figure 1H). Later, LRP1 was shown to directly associate with PDGFR-β to form a signal transduction complex (Newton et al., 2005; Muratoglu et al., 2010). As such PDGF signaling is influenced by LRP1 and *vice versa*. Recently, the group of May demonstrated that LRP1’s ICD also modulates the crosstalk between PDGF-BB and sphingosine-1 which is important for modulation of PDGF-BB induced cell migration and blood vessel maturation (Nakajima et al., 2014). The possible relevance for tumor angiogenesis is yet to be determined. LRP1 also affects angiogenesis among other things via is regulatory role in VEGF signaling. The complex of the angiogenic inhibitor thrombospondin-1 and VEGF is internalized via LRP1 (Greenaway et al., 2007).

## The Relevance of LRP1 for the Interaction Between Malignant Cells and the Tumor (Micro)Environment

Both LRP1 expressed in malignant cells themselves and LRP1 expressed in non-tumorous cells present in the tumor (micro)environment are relevant for modulation of the above described cancer-modifying pathways. These pathways are involved in processes like growth and survival of tumor cells, angiogenesis, extravasation of tumor cells, invasion and metastasis. The relative expression of LRP1, its ligands and co-receptors, irrespective whether expressed by the tumor cells themselves or other cells in the tumor (micro)environment determine the modifying role of LRP1 in these different, but linked processes, which may in fact result in opposing effects on cancer progression.

Montel et al. (2007) silenced LRP1 in tumor cells only and host LRP1 was left untouched. They observed the failure of metastatic foci to grow in the lungs from xenografts of CL16 cells in SCID mice thus illustrating the relevance of LRP1 expression in tumor cells themselves.

Also the importance of LRP1 expression in non-tumor cells in the tumor environment has been demonstrated. In the breast tumor microenvironment, it was reported that the pro-cath-D protease, highly secreted by tumor cells, may trigger mammary fibroblast outgrowth in a paracrine LRP1-dependent manner (Beaujouin et al., 2010). The molecular mechanism engaged appears atypical as pro-cath-D interacts with the extracellular part of LRP1 β-subunit mediating the inhibition of LRP1-regulated intramembrane proteolysis in mammary fibroblasts (Derocq et al., 2012; Laurent-Matha et al., 2012). Recently, Staudt et al. (2013) demonstrated that the recruitment of LRP1-deficient monocytes into subcutaneous and orthotopic pancreatic tumors were significantly increased. The secretion of chemokines by LRP1-deficient macrophages is enhanced (especially CCL3), resulting in an increased number of tumor-associated macrophages (TAM) in the tumor site. The authors provided evidence that the LRP1-deficient TAM collectively contribute to an increased VEGF amount into the tumor microenvironment, leading to increased tumor angiogenesis.

The aforementioned role of LRP1 in heparanase activation and uptake implicates a potential regulatory role for LRP1 in exosomes biogenesis. As reviewed elsewhere (De Toro et al., 2015), exosomes are nanovesicles secreted by various cell types, including cancer cells, that serve in cell–cell communication. They can be isolated from body fluids and are regarded potential biomarkers for diagnosis and prognosis. As recently shown, syndecan heparan sulfate (HS) proteoglycans and heparanase are involved in exosome production (Baietti et al., 2012; Roucourt et al., 2015). Trimming of HS chains on syndecan molecules by heparanase appears to affect the formation of multimeric complexes of syndecans, co-receptors and the intracellular adaptor protein syntenin triggering the generation of intraluminal vesicles in multivesicular bodies (MVBs), eventually resulting in the release of exosomes. Heparanase apparently does not only regulate secretion of tumor-cell derived exosomes, but also its composition and function (Thompson et al., 2013). As such, LRP1-mediated control on active heparanase availability could effect exosome production and function.

## Drug Delivery Across the Blood–Brain Barrier

**FIGURE 2:**
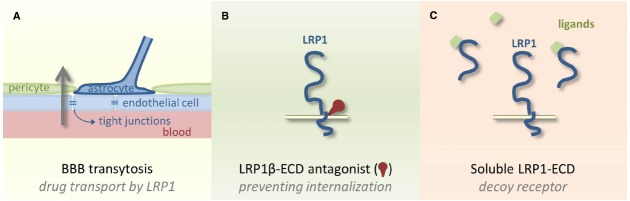
**Potential clinical applications of LRP1. (A)** Using LRP1 as a cargo receptor to sluice chemotherapeutic agents through the tightly controlled blood–brain barrier via transcytosis is undergoing clinical testing. **(B)** LRP1 antagonists to the ECD could prevent LRP1 internalization and catabolization. **(C)** The ECD of LRP1 could also be used as a soluble decoy receptor to capture LRP1 ligands and avoid ligand-receptor interaction.

Current studies on therapeutic strategies involving LRP1 focus on using it as a cargo receptor to treat brain metastases. The aforementioned expression of LRP1 in glioblastoma and other brain cancers (Yamamoto et al., 1997; Baum et al., 1998) or metastasis combined with LRP1’s expression at the BBB (Pflanzner et al., 2011) is crucial to this strategy. The capability of LRP1 to mediate transcytosis of a broad range of ligands through the BBB (Figure 2A) could be the long-awaited sluice for chemotherapeutic agents into the brain as BBB penetration is currently the Achilles’ heel in brain cancer therapies (Jovčevska et al., 2013). Uptake of paclitaxel through the BBB followed by endocytosis into tumor cells was shown to be increased after conjugating the taxane paclitaxel to a 19 amino acid sequence named angiopep-2 (Bertrand et al., 2011). This peptide was derived from the Kunitz domain, a known ligand of LRP1. A phase I clinical study showed that this conjugate (GRN1005) is well tolerated (Kurzrock et al., 2012; Drappatz et al., 2013). Therapeutic concentrations could be reached in the tumor and three patients where prior taxane therapy was unsuccessful showed partial response with GRN1005. After an initial phase II study, additional phase II studies are currently ongoing for patients with brain metastases from breast cancer and high grade glioma. Also other constructs are evaluated preclinically including an anti-HER2 antibody conjugated to angiopep-2 to treat brain metastasis from HER2 positive breast cancers (Regina et al., 2015). As demonstrated recently *in vitro* and in animal studies, angiopep-2 could also aid active transport of polymersomes through the BBB via LRP1 mediated trancytosis suitable for antibody delivery to the brain (Tian et al., 2015).

Apart from angiopep-2, also peptides containing a serine-arginine-leucine (SRL) sequence bind LRP1 and were recently shown to aid PAMAM nanoparticle transport across the BBB (Zarebkohan et al., 2015). These LRP1 targeted particles could become a valuable tool for non-invasive gene targeting to the brain.

Although highly challenging, developing strategies aiming at LRP1 targeting should be relevant in certain tumor microenvironments. We might consider new LRP1 antagonists targeting the extracellular part of the LRP1 β-subunit to avoid LRP1 itself being internalized and catabolized (Figure 2B). Another alternative could be to use the soluble LRP1-ECD as a decoy receptor to interfere with endocytic and signaling activities of cell-surface LRP1 (Figure 2C). The proof of concept exists for TIMP-3. Bound to LRP1-ECD, TIMP-3 becomes resistant to endocytosis and degradation and retains its inhibitory activity against metalloproteinases (Scilabra et al., 2013). LRP1 ligand-binding domains II and IV are probably the most critical regions that could serve as molecular and structural models for designing new therapeutic tools.

## Potential of LRP1 in Diagnosis and Prognosis

As discussed previously, in some cancer types, LRP1 expression was correlated with invasiveness, tumor stage, and even clinical outcome. However, although it has been suggested that LRP1 could be a potential biomarker (Meng et al., 2011), so far, there seems to be lots of variability and discussion. As mentioned before, LRP1 expression in cell cultures is also debatable as the *in vitro* conditions could affect LRP1 expression. Recent work on data from tumor samples identified LRP1 as a hub in a biomarker network for multi-cancer clinical outcome prediction (Martinez-Ledesma et al., 2015). This further illustrates the involvement and possible prognostic value of LRP1 in various cancers. Future large scale studies on patient samples could provide more insights and demonstrate the true relevance of LRP1 in diagnosis and prognosis of cancer.

## Conclusion

Via a diverse array of interactions LRP1 modulates various pathways involved in cancer. Especially its role in modifying the ECM could be crucial for tumor growth and metastasis. However, considering the sometimes contradicting studies LRP1 cannot be considered a master switch as some prototype oncogenes or tumor suppressor genes are. Rather, it acts as an interface to fine-tune various cancer-related pathways. Its effects appear to be dependent on both the tumor type and the tumor environment. This complicates LRP1 research and calls for good model systems that integrate the diverse set of LRP1 activities. These should answer the question whether LRP1 could be a valuable target for diagnosis, prognosis and therapeutics in cancer as well as other diseases.

### Conflict of Interest Statement

The authors declare that the research was conducted in the absence of any commercial or financial relationships that could be construed as a potential conflict of interest.
